# Reverse Iontophoretic Extraction of Skin Cancer-Related Biomarkers

**DOI:** 10.3390/pharmaceutics14010079

**Published:** 2021-12-29

**Authors:** Maxim Morin, Sebastian Björklund, Skaidre Jankovskaja, Kieran Moore, Maria Begoña Delgado-Charro, Tautgirdas Ruzgas, Richard H. Guy, Johan Engblom

**Affiliations:** 1 Department of Biomedical Science, Faculty of Health and Society, Malmö University, SE-205 06 Malmö, Sweden; sebastian.bjorklund@mau.se (S.B.); skaidre.jankovskaja@mau.se (S.J.); tautgirdas.ruzgas@mau.se (T.R.); 2 Biofilms—Research Center for Biointerfaces, Malmö University, SE-205 06 Malmö, Sweden; 3 Department of Pharmacy & Pharmacology, University of Bath, Claverton Down, Bath BA2 7AY, UK; km524@bath.ac.uk (K.M.); prsbd@bath.ac.uk (M.B.D.-C.); r.h.guy@bath.ac.uk (R.H.G.)

**Keywords:** tryptophan, kynurenine, tryptophan-to-kynurenine ratio, cancer-related biomarkers, non-invasive extraction, bicontinuous cubic liquid crystals

## Abstract

Non-invasive methods for early diagnosis of skin cancer are highly valued. One possible approach is to monitor relevant biomarkers such as tryptophan (Trp) and kynurenine (Kyn), on the skin surface. The primary aim of this in vitro investigation was, therefore, to examine whether reverse iontophoresis (RI) can enhance the extraction of Trp and Kyn, and to demonstrate how the Trp/Kyn ratio acquired from the skin surface reflects that in the epidermal tissue. The study also explored whether the pH of the receiver medium impacted on extraction efficiency, and assessed the suitability of a bicontinuous cubic liquid crystal as an alternative to a simple buffer solution for this purpose. RI substantially enhanced the extraction of Trp and Kyn, in particular towards the cathode. The Trp/Kyn ratio obtained on the surface matched that in the viable skin. Increasing the receiver solution pH from 4 to 9 improved extraction of both analytes, but did not significantly change the Trp/Kyn ratio. RI extraction of Trp and Kyn into the cubic liquid crystal was comparable to that achieved with simple aqueous receiver solutions. We conclude that RI offers a potential for non-invasive sampling of low-molecular weight biomarkers and further investigations in vivo are therefore warranted.

## 1. Introduction

Non-melanoma skin cancers (NMSCs), such as basal-cell carcinoma (BCC) and squamous cell carcinoma (SCC), are the most common forms of skin cancer [1]; in contrast, the melanoma-related skin cancers are less common, but more dangerous (due to their ability to spread to other organs), and have become one of the fastest-growing forms of the disease [2]. The most crucial factor for the higher incidence rate is increasing exposure to the UV radiation [3]. Clearly, therefore, the early detection of cancer at early stage is a crucial requirement to retard or stop progression and to increase long-term survival. The current gold standard for skin cancer diagnosis relies primarily on visual inspection of a lesion followed by tissue biopsy and staining. The accuracy of this approach depends on factors such as clinician experience and lesion characteristics [4], and is reported to vary between 49 and 81% [5,6,7,8,9]. For early-stage melanoma, the current gold standard diagnostic procedure has low specificity (<30%) and moderate sensitivity (~84%) [10]. To illustrate this point, benign nevus, a highly common and harmless lesion type, can be mistaken for cutaneous melanoma, resulting in unnecessary excision of a significant number of benign lesions [11]. Further, although tissue biopsies represent the standard method to diagnose skin cancer, they are invasive and associated with a risk of infection, and are expensive and typically involve long waiting times for patients [12,13]. It follows that the development of an alternative, or complementary, non-invasive method to detect early-stage skin cancer is urgently required. With higher specificity and sensitivity, such a method would help to reduce the number of unneeded biopsies and to justify when surgical intervention is warranted [4,11].

Skin cancer also affects the chemistry of the tissue, altering levels of certain endogenous substances and their metabolites [14]. In addition, in the case of actinic keratoses and Bowen’s disease, which can eventually develop into SCC [3], sustained inflammation is a precursor for cancer and the detection of inflammatory biomarkers can therefore provide an early warning of disease onset [1,15]. Indeed, our knowledge regarding biomarkers associated with inflammation and cancer (e.g., IL-6, IFN-γ, TNF-α, enzyme indoleamine-2, 3-dioxygenase (IDO), BRAF gene mutations) is continuously increasing [16,17,18,19,20]. The majority of these compounds, however, are macromolecules and, therefore, unable to be passively extracted from the viable epidermis across the stratum corneum (SC) [21,22]. On the other hand, the catabolism of the essential amino acid tryptophan (Trp) along the kynurenine pathway (KP) plays a crucial role in regulation of the immune response [23,24] and changes in the Trp/Kyn ratio are associated with several diseases including cancer [25,26,27]. The physicochemical properties of Trp and Kyn (Table 1), in particular their modest molecular weights, make them potentially suitable candidates for topical sampling but their polar nature raises questions as to whether sufficient amounts can be extracted within reasonable time periods to allow for subsequent quantification.

A possible solution to circumvent this problem is use of reverse iontophoresis, which can significantly enhance the transport of charged and polar substances across the skin through the application of a small electric current (<0.5 mA/cm^2^) [28]. Upon application of an electric field in iontophoresis, three contributions to the total flux of the target compound are possible [29]: (i) electromigration, whereby charged species are electrostatically repelled from an electrode of like polarity and attracted to one of the opposite charge [28]; (ii) electroosmosis, which is a net flow of solvent across the skin in the anode-to-cathode direction and results when the electric field is applied across the net negatively-charged skin (pI ~4.0 to 4.5) [28]; and (iii) passive diffusion, which for amino acids is significantly lower relative to electromigration and electroosmosis [30]. This minimally invasive technique has been shown effective for the monitoring of drugs [31], the constituents of natural moisturizing factor (NMF) [32,33,34,35], glucose [36,37], phenylalanine (to diagnose phenylketonuria [38], urea (as a biomarker of chronic kidney disease) [39], and prostaglandin E2 (a molecule associated with cutaneous inflammation) [40]. As most amino acids are zwitterionic at pH 7.4, electroosmosis towards the cathode on the skin surface is the primary mechanism by which they are extracted transdermally in reverse iontophoresis [28,32,34,41].

While reverse iontophoretic extraction has usually been performed with a buffer solution as the ‘receiver’ phase, the use of a patch, which can be easily applied to the skin, would be more practically convenient. As a result, patches comprising hydrogels have been widely investigated because of their biocompatibility, high water content, and ease of handling [42,43,44,45,46]. Another, potentially better ‘receiver’ could be a lipid-based patch. For example, glycerol monooleate (GMO) exhibits a versatile polymorphism in water and is associated with low toxicity and great biocompatibility and biodegradability [47]. In excess water, GMO forms a bicontinuous cubic phase (C_D_ double-diamond, crystallographic space group Pn3m) [47,48,49], with a highly interconnected network of water and lipid channels that can accommodate hydrophilic, hydrophobic, and amphiphilic compounds [50,51,52,53]. These water channels also make bicontinuous cubic phases particularly suitable for iontophoretic applications, as ions and small hydrophilic molecules can move freely inside the material. While this cubic phase has been tested as a vehicle for the iontophoretic delivery of salbutamol across the skin [54], no applications in reverse iontophoretic extraction have been reported.

The primary aim of this work, therefore, was to investigate in vitro whether reverse iontophoresis is a suitable technique to enhance the extraction of Trp and Kyn from the viable skin; it was a further objective to show that the extracted Trp/Kyn ratio obtained at the skin surface reflected that in the viable tissue. Secondary goals were (a) to evaluate the effect of receiver solution pH on extraction efficacy, and (b) to assess the performance of a lipid-based, liquid crystalline matrix as a receiver for the targeted iontophoretically sampled biomarkers. The long-term goal is a more accurate skin cancer diagnosis that permits unwarranted excision of benign lesions to be minimised.

## 2. Materials and Methods

### 2.1. Materials

Sodium chloride (NaCl) and sodium phosphate dibasic (Na_2_HPO_4_) were purchased from Fisher Scientific (Loughborough, UK); potassium phosphate monobasic (KH_2_PO_4_) was purchased from Sigma-Aldrich (Tokyo, Japan), HEPES (*N*-2-hydroxyethylpiperazine-*N*-2-ethanesulfonic acid) was obtained from Acros Organic (Geel, Belgium). Silver chloride (AgCl, metal basis > 99.99% purity) and silver (Ag) wire 1 mm Ø (>99.99% purity) were sourced from Sigma-Aldrich (Gillingham, UK). Amino acid l-tryptophan (Trp, ≥98% HPLC) was purchased from Sigma-Aldrich (Shanghai, China) and l-kynurenine (Kyn, metabolite of tryptophan, ≥98% HPLC) was from Sigma-Aldrich (Buchs, Switzerland). Glycerol monooleate (RYLO^TM^ MG 19 Pharma, Batch nr. 4010989490, monoglyceride content > 95% *w*/*w*, *M*_r_ = 356.6 g/mol) was kindly provided by Danisco Cultor (Brabrand, Denmark). PBS buffer (130.9 mM NaCl, 5.1 mM Na_2_HPO_4_ and 1.5 mM KH_2_PO_4_, pH of 7.4) and HEPES buffer (10 mM HEPES, 60 mM NaCl) were prepared in Milli-Q water (resistivity ≥ 18.2 MΩ·cm) and degassed by sonication for one hour prior to use. Methanol of HPLC grade was obtained from VWR international (Lutterworth, UK).

### 2.2. Preparation of Electrodes

The reversible Ag/AgCl electrode couple was preferred over platinum because it avoids electrolysis of water [29]. The electrodes were prepared by making a small loop at one end of a silver wire (~6 cm in length) that was then dipped into and coated with molten silver chloride (T = 475 °C). After cooling, anodes were prepared by conditioning the AgCl-coated loop overnight in a glass beaker (25 mL) against a platinum wire electrode (0.2 mm diameter, Sigma), at 0.3 mA with 50 mM NaCl as electrolyte and resulting in the formation of a silver layer on the outer surface (see Figure S1 in Supplementary Materials).

### 2.3. Skin Preparation

Pig skin (abdominal or from the inner ear surface, purchased from a local abattoir, was gently rinsed post-sacrifice under cold running water. The hair was trimmed and the skin was dermatomed to a final thickness of 750 µm (Dermatome, Integra LifeSciences, Plainsboro, NJ, USA) in accordance to the OECD guidelines [55,56] (Figure S2A–C in Supplementary Materials). For practical reasons, the skin samples were kept at −20 °C, wrapped individually in Parafilm^TM^, until use within three months. Prior to extraction experiments, skin was cut into 4 cm^2^ pieces and then left for 30 min to thaw completely. The integrity of each skin membrane was visually checked. Abdominal skin membranes were used for extraction experiments at pH 7.4 and pH 9, while skin from both the abdomen and the inner ear were used for extraction at pH 4. Control experiments and extraction into the cubic phase were performed using inner ear skin.

### 2.4. Preparation of Liquid Crystalline Cubic Phase as a Receiver

Firstly, 0.1 g of glycerol monooleate (GMO) was weighed into a 1.8 mL glass vial and melted at 45 °C by immersion in a water bath. After centrifugation at 1000× *g* for 5 min, the vials were placed in the freezer (−20 °C) for 30 min GMO allowed to crystallize. The vials were then brought to room temperature and 0.5 g (an excess) of Milli-Q water was added to each vial. A clear and highly viscous liquid crystalline cubic phase was formed within 2 days. Prior to use, each vial was examined between cross polarizers for any sign of anisotropy, which would imply that sample had not yet reached equilibrium.

### 2.5. Reverse Iontophoretic Extraction of Trp and Kyn

**Figure 1:**
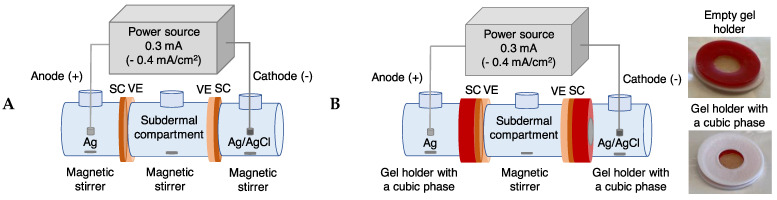
Graphical representation of the reverse iontophoresis experiments with anodal and cathodal receptor phases comprising either (**A**) an aqueous buffer (*n* = 5–12, see Table 2), or (**B**) a cubic phase in contact with an aqueous buffer (*n* = 3–5, see Table S7 in Supplementary Materials).

Reverse iontophoretic experiments were performed in horizontal side-by-side cells comprising anodal and cathodal receptor compartments (2 mL each), and a subdermal ‘donor’ compartment (3 mL) (Figure 1A). Two skin membranes were mounted in each cell such that the SC faced the anode and cathode receptor compartments while the dermal surfaces contacted the donor compartment. The exposed skin area was 0.79 cm^2^. In all experiments, the donor compartment contained 1 mM Trp and 1 mM Kyn in PBS (i.e., a subdermal ratio of Trp/Kyn equal to 1). The background electrolyte solution in the receptor compartments (anodal and cathodal) was 10 mM HEPES, 60 mM NaCl buffered at either pH 4.0, 7.4 or 9.0. All solutions were degassed by sonication prior to the experiments. Extraction experiments with a cubic phase were performed in a similar manner as shown Figure 1B with custom made gel holders with the same area as the side-by-side cells. The cubic phase volume in the gel holder was ~100 µL. After assembly of side-by-side cells, anode (Ag) and cathode (Ag/AgCl) electrodes were inserted in their respective receptor compartment solutions that were magnetically stirred throughout the experiment. Reverse iontophoretic extraction of Trp and Kyn was performed at room temperature (21.5 ± 0.7 °C) by passing a constant current of 0.3 mA (0.4 mA/cm^2^) for 6 h Yokogawa 7651 Programmable DC source (Yokogawa, Japan)—see Figure S2D,E in the Supplementary Materials.

In the extraction experiments into buffer, hourly 1 mL samples were withdrawn from the receptor compartments and replaced with an equal volume of fresh buffer solution (the current was stopped during sampling). At 6 h, the current was terminated and the donor compartment emptied. The post-iontophoretic passive extraction from the skin membranes was then allowed to continue for a further 18 h when final samples from the electrode chambers were taken. Extraction into the cubic phase followed the same sampling procedure for 6 h when the experiment was ended. The cubic phase was then collected from the gel holder using a flat spatula and transferred into a 1.7 mL Eppendorf tubes leaving as little residue as possible on the skin surface. Trp and Kyn were extracted from the cubic phase into 1 mL of Milli-Q water with shaking for 1 h. Subsequently, the suspension was vortexed for 1 min and the aqueous supernatant was collected for analysis. A small sample of the cubic phase was taken for SAXD examination to determine whether phase changes had occurred after reverse iontophoresis.

### 2.6. Passive Diffusion Extraction of Trp and Kyn

To quantify the enhancement of extraction induced by reverse iontophoresis, the experiments using buffer receptor solutions were repeated in an identical fashion except that no current was applied (*n* = 4). Only the buffered solution was used as a receiver.

### 2.7. Control Experiment

To correct for any potential extraction of endogenous Trp and Kyn from the porcine skin employed, the reverse iontophoresis experiments into buffer receptor solutions were repeated in an identical fashion except that no exogenous Trp and Kyn were included in the subdermal compartment solution and the experiment was terminated at 6 h (*n* = 6).

### 2.8. Analytical Method

All extraction samples were filtered (0.2 µm syringe filters, Minisart™, RC-15, Sartorius, UK) and frozen (−20 °C) for up to 24 h and then thawed and vortexed before analysis by HPLC-UV (Shimadzu LC-2010 A HT system, Buckinghamshire, UK). Chromatographic separation of Trp and Kyn was performed on a 250 mm × 4.6 mm C18 HiQ Sil column with particle size of 3 µm (Kromatech, Dunmow, UK) using gradient elution with mobile phase A comprising 10 mM NaH_2_PO_4_ (pH 2.8) and mobile phase B of 100% MeOH at a flow rate of 0.9 mL/min and a column temperature of 40 °C. The gradient profile, modified from previous work [57], involved: (a) 0.0–7.0 min, mobile phase B was fixed at 25%; (b) 7.0–11.0 min, phase B was gradually increased to 95%; (c) 11.0–15.0 min, phase B was held at 95%; and (d) 15.0–15.1 min, phase B was decreased to 25% and maintained for a further 1.9 min. The total run time was 17 min and the injection volume was 20 µL. Trp and Kyn were detected at their UV absorbance maxima, at 280 and 360 nm, respectively. Stock solutions of 20 mM of Trp and Kyn for calibration curve were prepared in Milli-Q water and were used within 24 h of preparation. Standards for the calibration curves were analysed in triplicate (0.78 to 100 µM, R^2^ > 0.999) on the same day as the experimental samples. Limit of detection (LoD) and limit of quantification (LoQ) were determined to 0.13 µM and 0.40 µM for Trp, and 0.16 µM and 0.49 µM for Kyn. The analytes were quantified by manual integration of the corresponding peaks using LabSolution software (Shimadzu, Kyoto, Japan).

### 2.9. Small Angle X-ray Diffraction (SAXD)

SAXD (Xeuss 3.0 SAXS/WAXS, Xenocs, Grenoble, France) was used for cubic phase characterization. All samples were measured in an ambient environment at 25 °C, with a temperature-controlled Peltier gel-holder stage using an O-ring as a spacer between two Kapton films (DuPont^TM^ Kapton^®^, 0.013 mm thickness, Goodfellow, England). The diffraction data were collected by Pilatus3 R 300K hybrid photon counting detector with a sample-to-detector distance of 800 mm. One-dimensional data were obtained by azimuthal averaging of the 2D-diffraction pattern and the scattering intensity was corrected for background and normalised to the direct beam. The exposure time was 20 min for each sample. The lattice parameter, a measure of the smallest repeat distance in the unit cell, was calculated to determine potential changes in the cubic phase.

### 2.10. Data Analysis and Statistics

Anodal, cathodal, and passive fluxes (nmol/cm^2^/h) of Trp and Kyn were directly calculated from the amounts extracted at each sampling interval. The cumulative amounts extracted at 6 h were used to determine the enhancement induced by reverse iontophoresis and expressed as the ratio (ER) of that extracted with current to that extracted passively. When appropriate, the resulting data were represented as mean ± standard error of the mean (SEM). All statistical analyses were performed using RStudio (Version 1.3.1093) [58], and the level of statistical significance was fixed at α ≤ 0.05.

## 3. Results

### 3.1. Reverse Iontophoretic Extraction of Trp and Kyn at pH 7.4

**Figure 2:**
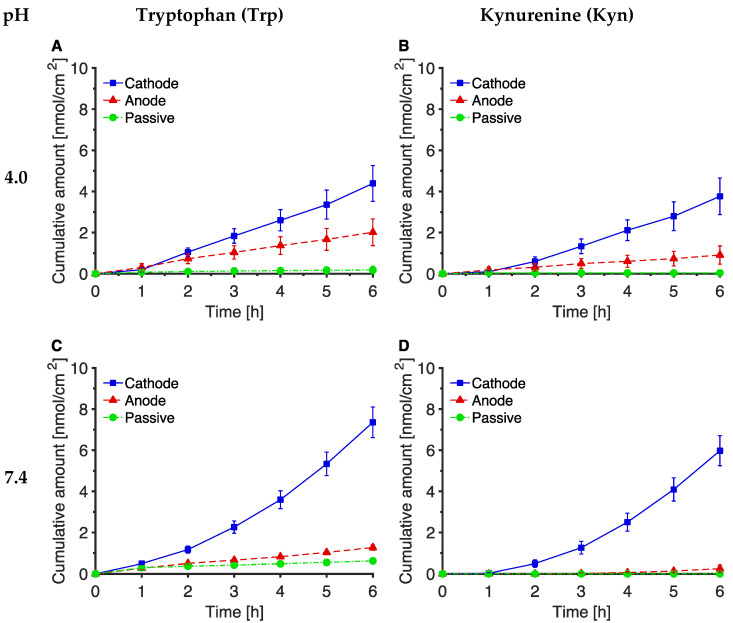

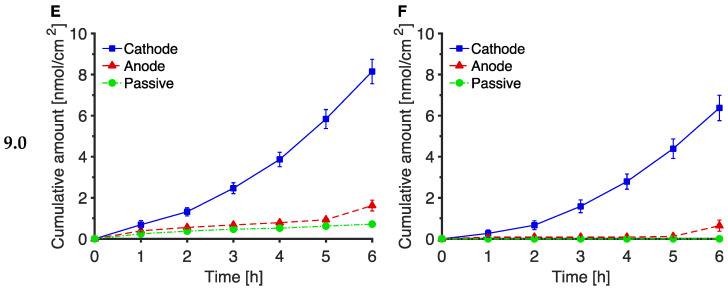
Cumulative amounts of Trp (**A**, **C**, and **E**) and Kyn (**B**, **C**, and **F**) extracted passively and by reverse iontophoresis as a function of time at three different receptor solution pH values: pH 4.0 (**A,B**), pH 7.4 (**C,D**), and pH 9.0 (**E,F**). The data points show the mean ± SEM (*n* = 4–12, see Table 2).

The passive and reverse iontophoretic cumulative extractions of Trp and Kyn, as a function of time, are shown in panels C and D, respectively, of Figure 2, and a summary of these data is in Table 2. Additional data are shown in Figures S5 and S6 and Table S1.

At pH 7.4 (i.e., when there is no pH gradient across the skin), the passive extraction of Trp was detectable at most times and, by 6 h, the cumulative amount reaching the receptor had reached 0.62 (± 0.13) nmol/cm^2^. For Kyn, on the other hand, the level diffusing passively across the skin was insufficient to be quantifiable with the assay used. Both Trp and Kyn extraction to the cathode were significantly enhanced (relative to passive diffusion) by reverse iontophoresis, a difference that was already clear after 2 h or less of current passage; the cumulative amounts of Trp and Kyn extracted were 7.4 (± 0.8) and 6.0 (± 0.7) nmol/cm^2^, respectively, and were not significantly different from one another. In contrast, reverse iontophoretic extraction to the anode was essentially no different than passive diffusion for either Trp or Kyn.

The deduced flux profiles from Figure 2 (panels C and D) are shown in the corresponding panels of Figure S7 in the Supplementary Materials (a summary of the data is also in Figure S8 and Table S2). The cathodal fluxes increased steadily over the 6 h of extraction (by which time the Trp and Kyn fluxes were 2.0 (± 0.2) and 1.9 (± 0.2) nmol/cm^2^/h, respectively) while that of Trp to the anode appeared to reach a relatively constant, but an order of magnitude lower, level of about 0.2 nmol/cm^2^/h. The passive fluxes of both compounds and the anodal flux of Kyn were even smaller and sometimes unquantifiable.

### 3.2. The Effect of pH on Trp and Kyn Extraction by Reverse Iontophoresis

Reverse iontophoretic extraction of Trp for 6 h into a pH 4 receiver solution at the cathode was significantly smaller (by ~40%) than that at pH 7.4 (Figure 2A,B and Table 2). For Kyn, the lower pH did not result in a statistically significant difference. Anodal extraction of both analytes was also insensitive to the pH change from 7.4 to 4. With electrode receiver solutions at pH 9, no significant differences in the reverse iontophoretic extraction (to anode or to cathode) of either Trp or Kyn were observed relative to the values at pH 7.4 (Figure 2C,D and Table 2). Finally, passive extraction of Trp at pH 4 and pH 9 was indistinguishable from that at pH 7.4, while that of Kyn remained unquantifiable at both the higher and lower pHs considered (Figure 2A,B and Table 2).

The deduced flux profiles at pH 4 and pH 9 from Figure 2 (panels A, B and E, F, respectively) are shown in the corresponding panels of Figure S7 in the Supplementary Materials (and a summary of the data is also in Tables S2 and S3). The average fluxes deduced from the last two measurements of the cumulative cathodal extractions (i.e., those amounts at 5 and 6 h) at pH 4 were significantly smaller than those at pH 7.4 and pH 9, for both Trp and Kyn. Otherwise, the changes in the pH of the receiver solution had no other significant effect on extraction of the two analytes.

### 3.3. Extraction of Endogenous Trp and Kyn

**Figure 3:**
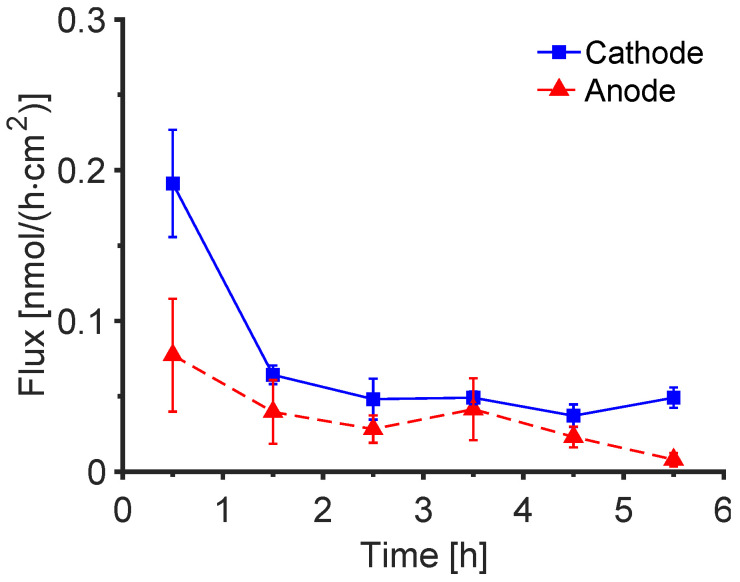
Flux of endogenous Trp extracted by reverse iontophoresis into electrode receiver solutions at pH 7.4. The data are presented as mean ± SEM (*n* = 6). Corresponding cumulative amount is shown in Figure S9 in Supplementary Materials.

Control experiments with a ‘placebo’ subdermal solution containing no exogenous Trp or Kyn revealed no quantifiable extraction of the latter to either cathode or anode after 6 h of reverse iontophoresis. However, extraction of endogenous Trp was detected at both electrodes with more of the compound found at the cathode as expected, see Figure 3. The highest extraction flux was observed in the first hour after which the rate decreased to ~0.05 nmol/cm^2^/h or less by 6 h. It follows that after 1–2 h of reverse iontophoresis, therefore, the contribution of endogenous Trp to the total amount extracted when the subdermal compartment contained the amino acid at a concentration of 1 mM was on the order of 3% or less.

### 3.4. Post-Iontophoretic Passive Extraction of Trp and Kyn

**Figure 4:**
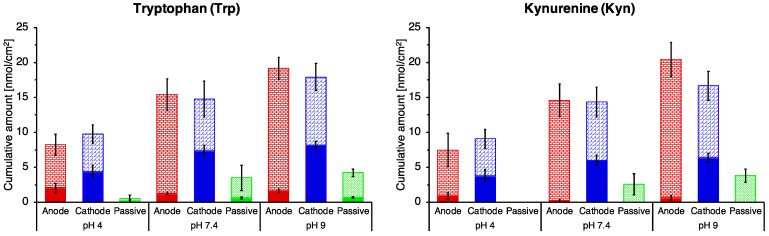
Cumulative anodal, cathodal and passive extraction of Trp and Kyn during 6 h of reverse iontophoresis (filled bars) and during a subsequent (no-current) period of 18 h (stippled bars). Data presented are mean ± SEM; *n* = 4–11, see Table S4 for detail.

Figure 4 (and Figure S10 and Table S4 in the Supplementary Materials) summarise the results from the experiments in which the ‘release’ of Trp and Kyn from the skin 18 h after a 6 h period of reverse iontophoresis was determined. It is apparent from these data that the amounts of the two analytes taken up (or partitioned) into the skin from the subdermal compartment during the 6 h extraction period were essentially the same regardless of the polarity of the electrode in the receiver compartment. However, the fraction of that quantity extracted into the receptor solution by the 6 h of current passage was clearly greater at the cathode than at the anode. This difference was then dissipated over the subsequent 18 h passive ‘release’ period. It is also evident that relative to the no-current passive control, iontophoresis enhanced the overall uptake of Trp and Kyn into the skin independent of electrode polarity. The pH of the receiver solution had only a modest effect, in line with the results reported above for iontophoretic extraction alone.

### 3.5. Reverse Iontophoretic Extraction into a Semisolid Matrix

**Figure 5:**
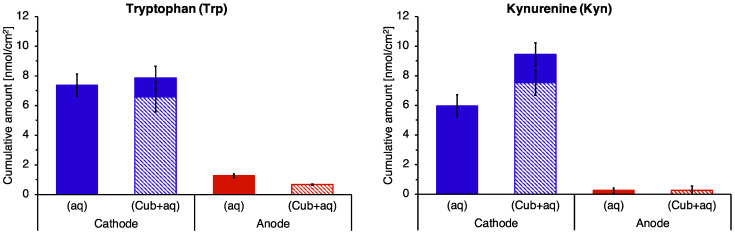
Cumulative Trp and Kyn extraction in 6 h of reverse iontophoresis into the cubic matrix (Cub+aq) and compared to that when an aqueous pH 7.4 solution was used as before. The data presented are mean ± SEM (*n* = 3 and 5 for extraction into the cubic matrix at anode and cathode, respectively; *n* = 6 for extraction into aqueous solution).

The cumulative reverse iontophoretic extraction of Trp and Kyn into semi-solid GMO cubic phase matrix in 6 h is shown in Figure 5 and compared with the results reported earlier when an aqueous, pH 7.4 buffer was used as the receiver medium; the data are also summarised in Table S7 in the Supplementary Materials. Successful and comparably efficient extraction into the cubic phase was achieved with, once more, significantly preferential electrotransport of the analytes to the cathode. The results show that Trp and Kyn were drawn into and eventually through the semi-solid matrix in the course of the extraction process.

Small-angle X-ray diffraction was employed to determine whether the cubic phase stability or structure (e.g., a phase change) was affected by the conditions of the extraction experiment. This involved examination of samples exposed in four specific scenarios: (a) a fully aqueous-swollen cubic phase (the control); (b) a cubic phase associated with the cathode during 6 h of iontophoresis but with no exogenous Trp and Kyn present; (c) the control cubic phase doped with 1 mM Trp and 1 mM Kyn, i.e., concentrations that are much higher than normal physiological levels [59], and substantially greater than those achieved in the extraction experiments; and (d) cubic phases collected from the anode and cathode receiver compartments after a 6 h reverse iontophoretic extraction experiment with 1 mM Trp and 1 mM Kyn initially present subdermally. The resulting diffraction patterns are provided in Supplementary Materials Figure S11. GMO is known to form a bicontinuous cubic phase (Pn3m) at room temperature in excess water with a lattice parameter of about 92–96 Å, (depending in the purity) [50,60,61,62]; here, the value measured was 93.5 Å. Exposure to an iontophoretic current of 0.5 mA/cm^2^ caused the lattice parameter to decrease to 91.0 Å, a difference unlikely to be due to any noticeable structural change. Doping the cubic phase with the two analytes at a concentration of 1 mM shifted the lattice parameter to 95.2 Å, again a rather small change relative to the control. Finally, the combination of current and the concomitant uptake of Trp and Kyn into the matrix resulted in the lattice parameter increasing to 97.1 Å, but, again, such a change is considered modest at best and inconsequential with respect to the stability of the cubic phase.

### 3.6. The Trp/Kyn Ratio

**Figure 6:**
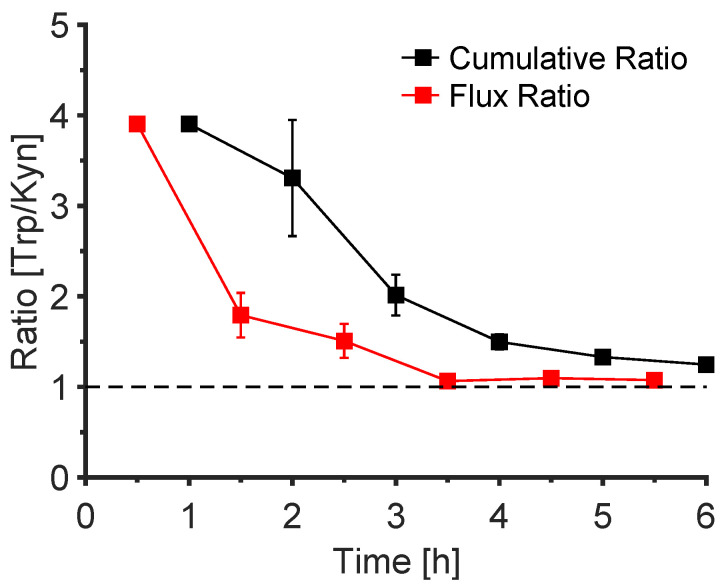
Trp/Kyn ratios of the cumulative amounts extracted (black) and the corresponding fluxes (red) to the cathode as a function of time when the receiver medium was an aqueous pH 7.4 buffer. Data are presented as mean ± SEM (*n* = 1 for the first hour, *n* = 4 for the second hour, and *n* = 6 thereafter).

To assess whether reverse iontophoresis has potential for the extraction and detection of a compound or compounds that may be useful biomarkers of diseases such as cancer, the Trp and Kyn data acquired were assessed in terms of the ratios of (i) the cumulative amounts extracted, and (ii) the fluxes across the skin. For this range-finding study, the subdermal (‘donor’) concentrations of the two analytes were fixed at 1 mM to provide a ratio of 1 against which to compare the relative extraction efficiencies. Figure 6 (also Figure S12) illustrates the evolution over time of the ratios of Trp to Kyn cumulative amounts extracted and of their iontophoretic fluxes to the cathode when the receiver medium was a pH 7.4 aqueous buffer. The initial ratios were high (~4) due to the effect of endogenous Trp extraction described previously (Figure 4) but, within a few hours, the ratios decreased and approached one. This behaviour was consistently seen when the pH of the receptor solution was changed to either four or nine (Table 2 and Table S2), or when the semi-solid cubic phase was used (Table S7).

## 4. Discussion

Reverse iontophoresis significantly enhanced, by an order of magnitude or more, the extraction of Trp and Kyn across the skin relative to passive diffusion (Figure 2 and Figure S5; Table 2) and was significantly greater in the anode-to-cathode direction compared to the reverse. For these zwitterionic compounds with pH 7.4 aqueous buffer solutions on both sides of the skin, this outcome is entirely consistent with the direction of electroosmotic flow which is induced when an electric field is imposed across a net negatively-charged membrane like the skin. The results are, furthermore, completely consistent with previous work that has used reverse iontophoresis to extract and identify the constituent amino acids in the skin’s natural moisturising factor [33,35].

The extraction flux of Trp was initially higher than that of Kyn before reaching something close to parity after 6 h of reverse iontophoresis (Figure S7). This was due to the presence of endogenous Trp [63] in the skin that was fairly quickly depleted (within a few hours) after current passage was initiated. The level of endogenous Trp deduced from these experiments was in good agreement with previous estimates reported in the literature [41,57] from both iontophoretic and passive extraction. No conclusions about the presence of endogenous Kyn could be drawn in the present (and earlier [57]) work however, as the analytical method for this compound was insufficiently sensitive.

The effect of changing the pH of the receptor, electrode solutions to either pH 4 or 9 was examined. Although the degree of ionization of Trp and Kyn was not substantially altered from their predominantly zwitterionic nature (Figures S3 and S4), the net negative charge on the skin was expected to be affected at the lower pH given that the isoelectric point of the membrane was estimated to be 4.0–4.5 [29]. A lesser charge on the skin would be reflected in reduced electroosmotic flow and this was supported by a significant decrease in Trp extraction in terms of both the cumulative amount and the terminal flux at 6 h (Figure 2 and Table 2); no significant difference, however, was observed for Kyn. The extraction of both Trp and Kyn in the anode-to-cathode direction remained, nonetheless, significantly greater than that in the opposite sense; this implies that the skin had not completely lost its net negative charge, a consequence perhaps of the fact that the lower pH was present on only one side of the membrane and not both (the subdermal compartment always being held at pH 7.4). Increasing the pH of the receptor, electrode solutions to 9 had no significant impact on the extraction of either Trp or Kyn, suggesting that any increase in electroosmosis had been offset by the analytes gaining any partial negative charge (Figures S3 and S4).

Experiments, in which the ‘release’ of Trp and Kyn from the skin was measured after 6 h of iontophoresis, revealed that uptake of the analytes from the subdermal compartment during current passage was essentially the same at both electrodes. That is, the sum of that extracted into the receiver, electrode solution in the 6 h of current passage, plus that which was released from the skin in the subsequent 18-h period, was the same at anode and cathode; however, the relative proportions of these two contributions were different (Figure 4 and Figure S10 and Table S4). At the cathode, more of the analytes were extracted during reverse iontophoresis than at the anode, but then less was released in the following 18 h. The reverse was the case at the anode. This pattern of behaviour was independent of the pH of the receiver, electrode solution; although, as discussed above, extraction efficiency was generally lower at pH 4 compared to pH 7.4 and pH 9.

An alternative and potentially more convenient matrix for reverse iontophoretic extraction was briefly examined using a cubic lipid (GMO) phase. Overall, it was found that extraction efficiency was similar to that achieved with a simple aqueous solution and, importantly, that the lipid matrix retained its stability and structural integrity under the conditions of the reverse iontophoretic process. Relatively small changes in the SAXD-determined lattice parameters were measured, the most noticeable of which were most likely due, it is believed [62], to the incorporation of the hydrophilic Trp and Kyn (and possibly other skin components) into the lipid bilayer.

A final observation from the research presented here is that the reverse iontophoresis of Trp and Kyn produced the consistent result that the ratio of the extracted amounts of the two analytes reflected that in the subdermal compartment (at least, once the endogenous Trp had been depleted as explained above). Certain caveats to this positive outcome must, however, be noted. For example, the coincidence of the ratios (i.e., that they were both ~1) may be a reflection of the similarity of the physicochemical properties of Trp and Kyn (Table 1); this will not always be the case if, for instance, the two compounds differ appreciably in terms of water solubility, or molecular weight, or if their net charges (or lack thereof) are not the same. This point is reinforced by the fact that the normal healthy physiological concentrations of Trp and Kyn are 71.1 and 2.3 μM, respectively, i.e., a ratio of Trp/Kyn = 30.9 [59]. Key questions to be posed, therefore, are the extent to which this ratio changes during the onset of a disease, such as cancer, and whether there are analytical approaches of sufficient sensitivity available to detect when a healthy ration transitions to one that is reliably diagnostic of the need for clinical intervention. Further work is clearly required to address these important issues, and to broaden the search for additional biomarkers that may be suitable for reverse iontophoretic extraction (e.g., those with a net charge at a physiological pH and/or that are present in the skin at concentrations that change substantially with the onset of a disease pathology).

## 5. Conclusions

The extraction of Trp and Kyn was significantly enhanced by reverse iontophoresis, and particularly in the anode-to-cathode direction. Once the endogenous Trp present in the skin had been depleted, the quantities of, and the fluxes at, which the two compounds were extracted were similar. The Trp/Kyn ratio of these metrics closely matched the ratio of the analytes’ subdermal concentrations (i.e., ~1). Changing the pH of the aqueous receptor phase, into which Trp and Kyn were extracted, from 7.4 to 9 had a negligible effect on electrotransport, while lowering to pH 4 resulted in a modest decrease in efficiency. A completely hydrated, semi-solid, bicontinuous lipid cubic phase performed well as an alternative and more practical (i.e., structurally stable) receptor into which Trp and Kyn could be extracted. It appears that further investigation of reverse iontophoresis as a relatively non-invasive method, with which small biomarker molecules of inflammation and cancer can be sampled, is warranted.

## Figures and Tables

**Table 1:** Physicochemical properties of two cancer related biomarkers.

Analyte	Structure	Mw [g/mol]	Log*D* *
Tryptophan (Trp)	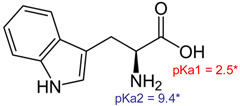	204.22	−1.1 at pH 4.0 −1.1 at pH 7.4 −1.2 at pH 9.0
Kynurenine (Kyn)	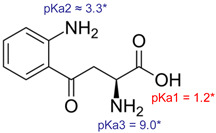	208.21	−2.0 at pH 4.0 −1.9 at pH 7.4 −2.2 at pH 9.0

* Theoretically determined values from ChemAxon software

**Table 2:** Cumulative amounts of Trp and Kyn extracted by reverse iontophoresis and passive diffusion after 6 h and at three different receiver solution pH values. The data presented are mean ± SEM.

pH ^1^	Analyte	Cumulative Amount Extracted (nmol/cm ^2^)			Trp/Kyn Ratio ^2^	ER ^3^	
		at Anode	at Cathode	Passively		Anode	Cathode
4.0	Trp	2.0 ± 0.7 (*n* = 12)	4.4 ± 0.9 (*n* = 10)	0.2 ± 0.2 (*n* = 4)	1.4 ± 0.2 (*n* *=* 9)	11	24
	Kyn	0.9 ± 0.4 (*n* = 12)	3.8 ± 0.9 (*n* = 10)	<LOQ (*n* = 4)		-	-
7.4	Trp	1.3 ± 0.1 (*n* = 6)	7.4 ± 0.8 (*n* = 6)	0.6 ± 0.1 (*n* = 4)	1.3 ± 0.3 (*n* = 6)	2	12
	Kyn	0.3 ± 0.2 (*n* = 6)	6.0 ± 0.7 (*n* = 6)	<LOQ (*n* = 4)		-	-
9.0	Trp	1.6 ± 0.3 (*n* = 5)	8.2 ± 0.6 (*n* = 6)	0.7 ± 0.1 (*n* = 4)	1.3 ± 0.6 (*n* = 6)	2	11
	Kyn	0.6 ± 0.3 (*n* = 5)	6.4 ± 0.7 (*n* = 6)	<LOQ (*n* = 4)		-	-

^1^ pH of the receptor solution; the subdermal solution pH was always 7.4. ^2^ Trp/Kyn ratio of the cumulative amounts extracted at the cathode after 6 h of current application. ^3^ ER = ratio of the cumulative amount of analyte extracted at the cathode by reverse iontophoresis to that measured passively after the same period of time.

## Data Availability

All data are presented within the manuscript and Supplementary Materials.

## Supplementary Materials

The following are available online at https://www.mdpi.com/article/10.3390/pharmaceutics14010079/s1, Figure S1: preparation of electrodes for iontophoresis experiments, Figure S2: Preparation of skin membranes and reverse iontophoretic experimental setup, Figures S3 and S4: Charge distribution profiles for Trp and Kyn, Figure S5: Box plots showing the variation in the cumulative amounts of Kyn and Trp, Figure S6: Cumulative amounts of Kyn and Trp extracted after 6 h of reverse iontophoresis, Figure S7: Reverse iontophoretic and passive fluxes of Trp and Kyn, Figure S8: Reverse iontophoretic extraction flux of Trp and Kyn after 6 h, Figure S9: The cumulative amount of endogenous Trp extracted by reverse iontophoresis over 6 h, Figure S10: A subsequent (18 h) post-iontophoretic passive (no-current) extraction of Trp and Kyn, Figure S11: X-Ray diffraction patterns for the fully swollen cubic phase (GMO/H_2_O), Figure S12: Trp/Kyn ratios, Table S1: The effect of pH on the cumulative amounts of Trp and Kyn extracted after 6 h of reverse iontophoresis, Table S2: Passive and reverse iontophoretic extraction flux of Trp and Kyn, Table S3: Comparison between anode and cathode flux of Trp and Kyn after 6 h of reverse iontophoresis, Table S4: Amounts of Trp and Kyn ‘released’ from the skin in the 18-h period following 6 h of reverse iontophoretic or passive extraction, Table S5: Comparison between the post-iontophoresis amounts of Trp and Kyn ‘released’ into the anode and cathode receptor solutions at different pHs, Table S6: Post-iontophoresis anode-to-cathode (A/C) comparisons in the amounts of Trp and Kyn ‘released’ and their corresponding fluxes at different pHs, Table S7: Cumulative amounts of Trp and Kyn extracted by reverse iontophoresis into a cubic phase.
