# Supplementary material for: Reverse Iontophoretic Extraction of Skin Cancer-Related Biomarkers
Source: Pharmaceutics. 2021 Dec 29;14(1):79. doi: 10.3390/pharmaceutics14010079 (PMC8778044; doi:10.3390/pharmaceutics14010079)
Supplement: Supplementary file 1 [file pharmaceutics-14-00079-s001.zip › pharmaceutics-1503283-supplementary.pdf]

pharmaceutics

MDPI

Supplementary Materials: Reverse iontophoretic extraction of skin cancer-related biomarkers

Maxim Morin, Sebastian Björklund, Skaidre Jankovskaja, Kieran Moore, Begoña M. Delgado-Charro, Tautgirdas Ruzgas, Richard H. Guy and Johan Engblom

1. Preparation of the Electrodes, Skin Membranes and Revere Iontophoretic Setup

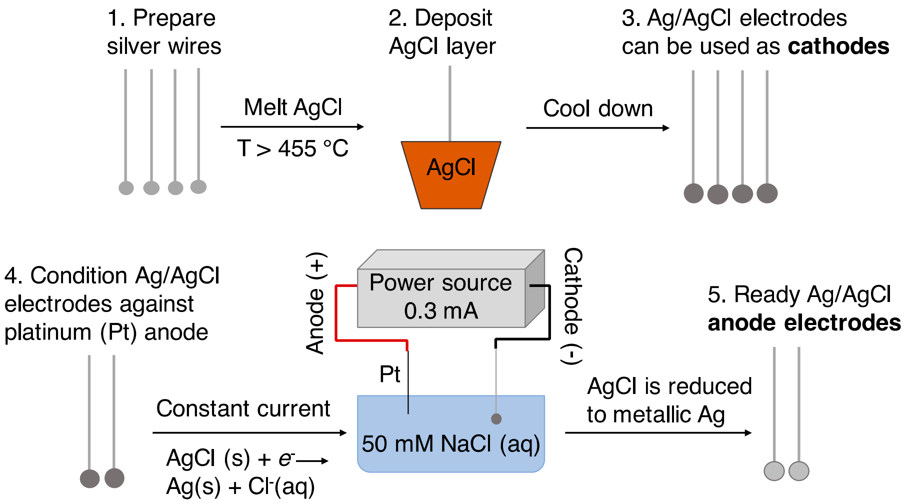

Figure S1. Schematics of the preparation of electrodes for iontophoresis experiments.

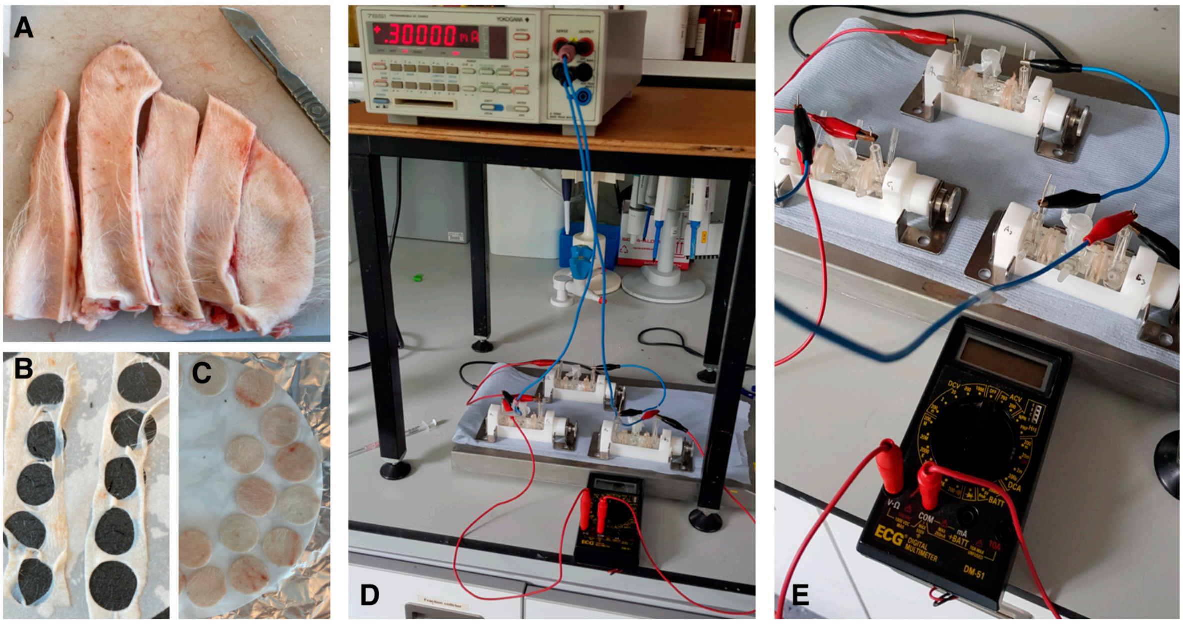

Figure S2. Preparation of skin membranes from inner surface of pig ear (**A**–**C**) and reverse iontophoretic experimental set up (**D**–**E**).

2. Charge Distribution of Tryptophan and Kynurenine as a Function of pH

It is important to know the charge on the molecules of interest, as this is an obviously key determinant of the direction of extraction. Charge distribution profiles for tryptophan

(Trp) and kynurenine (Kyn) obtained with ChemAxon software are shown in Figure S3 and Figure S4.

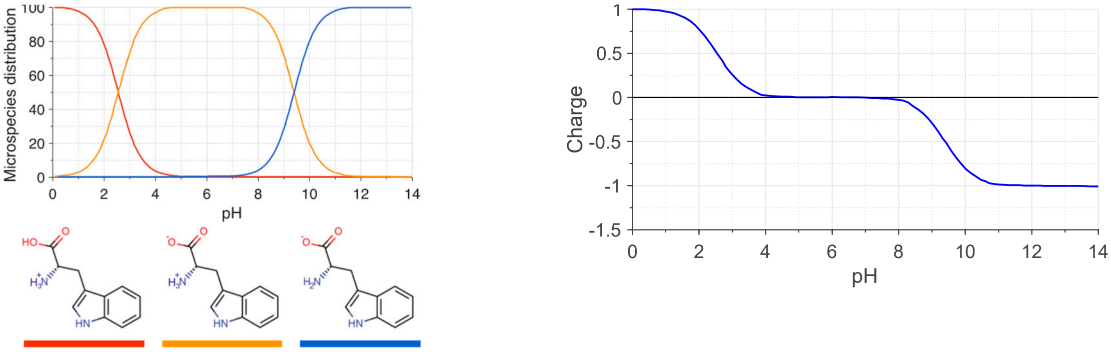

Figure S3. Charge distribution profile for tryptophan (Trp).

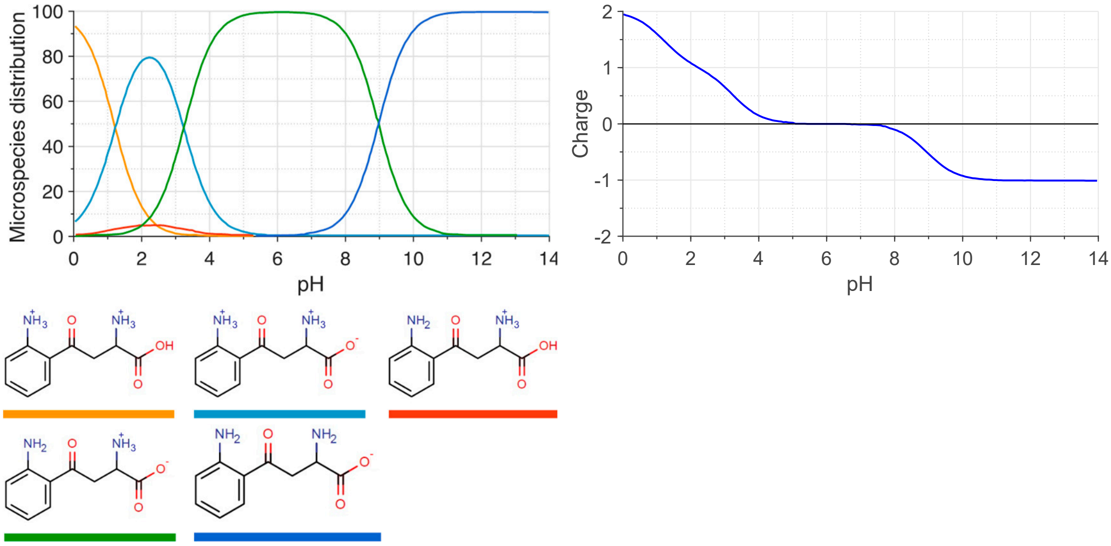

Figure S4. Charge distribution profile for kynurenine (Kyn).

3. Cumulative Amounts of Trp and Kyn Extracted by Reverse Iontophoresis

Tryptophan and its metabolite kynurenine were extracted across the skin membrane by reverse iontophoresis and passive diffusion. The current density during reverse iontophoresis was 0.4 mA/cm^2. Extraction experiments were conducted over 6 h at different receptor solution pH (i.e., 4.0, 7.4 and 9.0). The spread in the cumulative amounts of Trp and Kyn extracted after 6 h of reverse iontophoresis are shown in the box plots in Figure S5, and as the averages ± SEM in Figure S6. The variation in the data was smaller in extraction experiments performed at pH 7.4 and pH 9, compared to pH 4. The data obtained from extractions at the cathode were more normally distributed compared to the anode data.

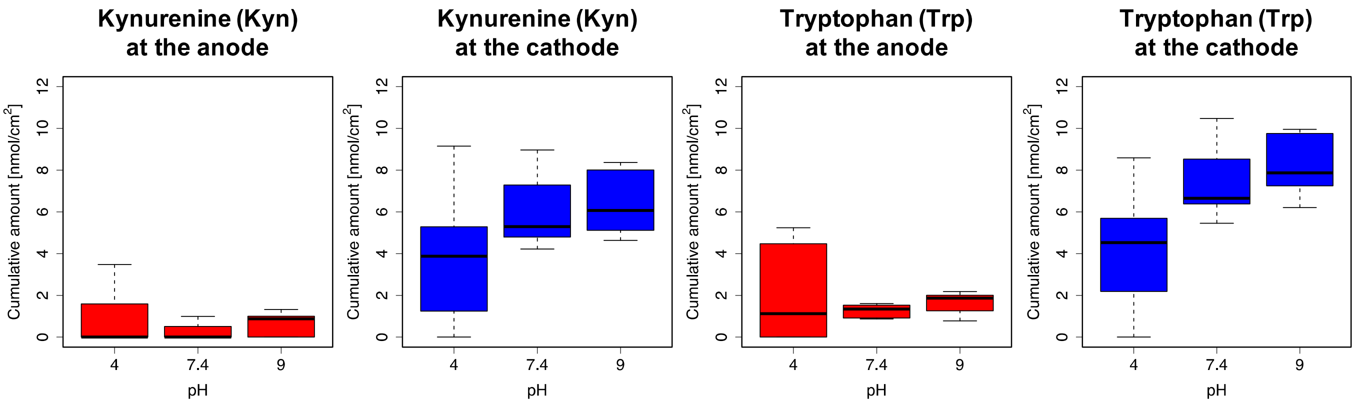

Figure S5. Box plots showing the variation in the cumulative amounts of kynurenine (top) and tryptophan (bottom) extracted at the anode (red) and at cathode (blue).

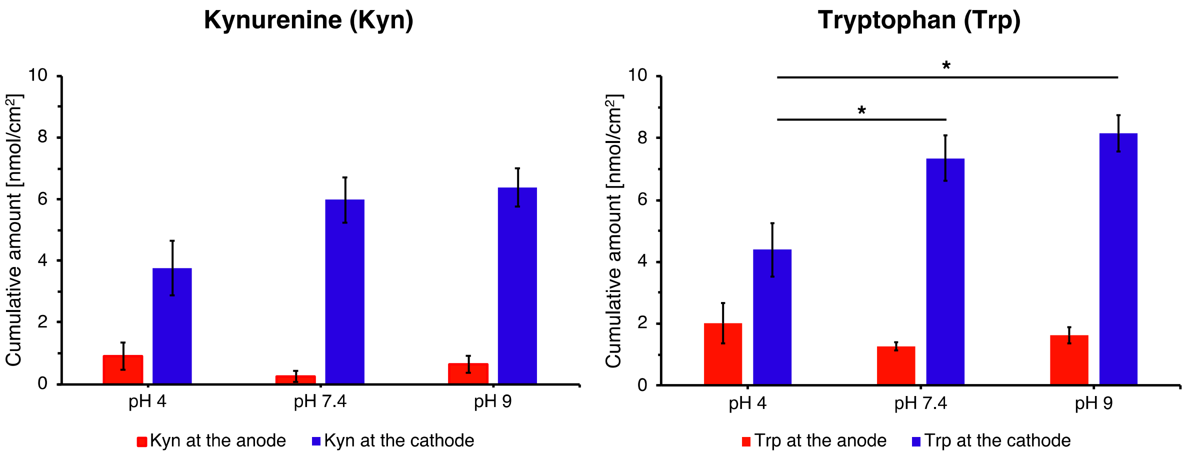

Figure S6. Cumulative amounts of kynurenine and tryptophan extracted after 6 h of reverse iontophoresis at the anode (red) and at the cathode (blue). Data show mean ± SEM (at pH 4, n_{anode} = 12, $n_{cathode}$ = 10; at pH 6, n_{anode} = 6, $n_{cathode}$ = 6; at pH 9, n_{anode} = 5, $n_{cathode}$ = 6). The significance levels: *p < 0.05, **p < 0.01, ***p < 0.001.

No significant differences were observed in the amounts of analytes extracted at different pH values towards the anode, whereas pH had a significant effect on Trp extraction at the cathode (that at pH 7.4 and at 9.0 being significantly higher than the value at pH 4.0) (Table S1).

Table S1. The effect of pH on the cumulative amounts of Kyn and Trp at the anode and cathode after 6 hours of reverse iontophoresis (ANOVA + Tukey's multiple comparison test). The significance levels: . p < 0.1, * p < 0.05, ** p < 0.01, *** p < 0.001.

pH	Kynurenine at the Anode			Kynurenine at the Cathode		
	t value	Pr(>|t|)	Significance	t value	Pr(>|t|)	Significance
7.4 − 4.0 == 0	−1.11	0.52	n.s.	1.88	0.17	n.s.
9.0 − 4.0 == 0	−0.43	0.91	n.s.	2.22	0.09	.
9.0 − 7.4 == 0	0.54	0.85	n.s.	0.31	0.95	n.s.
**pH**	**Tryptophan at the anode**			**Tryptophan at the cathode**		
	t value	Pr(>|t|)	Significance	t value	Pr(>|t|)	Significance
7.4 − 4.0 == 0	−0.88	0.66	n.s.	2.56	< 0.05	*
9.0 − 4.0 == 0	−0.44	0.90	n.s.	3.25	0.01	*
9.0 − 7.4 == 0	0.34	0.94	n.s.	0.613	0.81	n.s.

4. Iontophoretic and Passive Flux Profiles of Trp and Kyn

The instantaneous flux profiles of Trp and Kyn (plotted at the mid-point of the collection intervals) are shown in Figure S7.

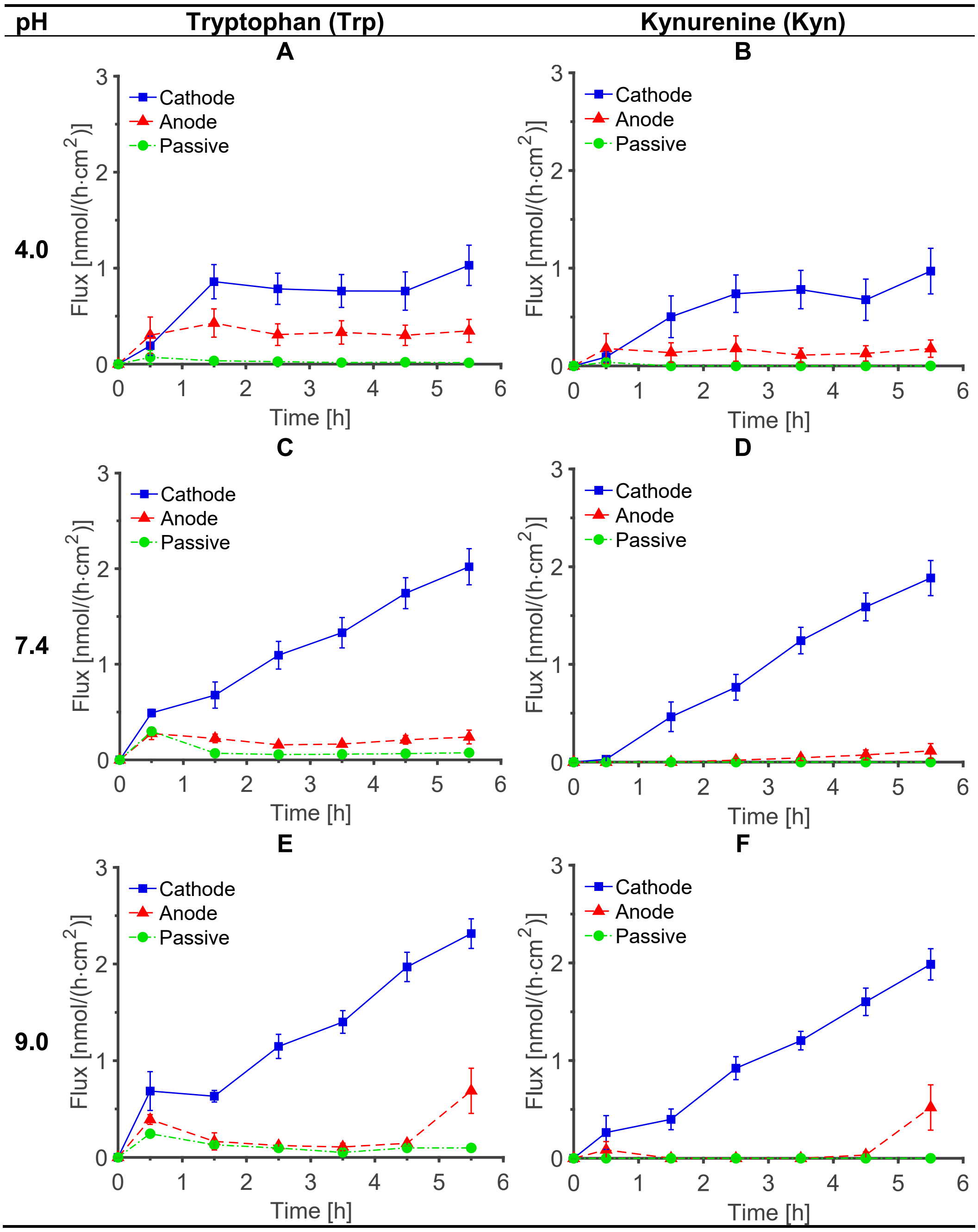

Figure S7. Reverse iontophoretic and passive flux profiles of tryptophan (left) and kynurenine (right) at pH 4.0 (**A, B**), pH 7.4 (**C, D**) and pH 9.0 (**E, F**). Extraction towards the cathode (blue squares) is compared to that at the anode (red triangles) and to passive diffusion (green circles). Each data point represents mean ± SEM (*n* = 4-12, see Table S2 for detail).

The average fluxes at the end of 6 h of reverse iontophoresis are in Figure S8 and Table S2.

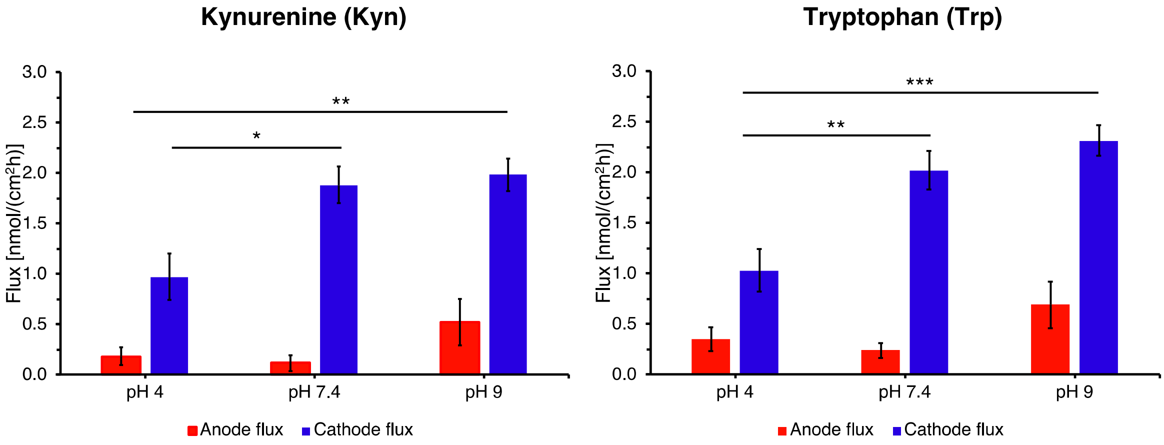

Figure S8. Reverse iontophoretic extraction flux (after 6 h) of kynurenine (red) and tryptophan (blue) at the anode (empty bars) and at the cathode (filled bars). The data represent mean ± SEM at pH 4 (at pH 4: n_{anode} = 12, $n_{cathode}$ = 10; at pH 6, n_{anode} = 6, $n_{cathode}$ = 6; at pH 9, n_{anode} = 5, $n_{cathode}$ = 6). The significance levels: * $p < 0.05$, ** $p < 0.01$, *** $p < 0.001$.

Table S2. Passive and reverse iontophoretic (at anode and at cathode) extraction flux of Trp and Kyn (mean ± SEM).

pH[1]	Analyte	Flux Into Electrode Receiver Solutions[2] [nmol/(h·cm²)]			Trp/Kyn Ratio[3]
		at anode	at cathode	passively	
4.0	Trp	0.35 ± 0.12 (*n* = 12)	1.03 ± 0.21 (*n* = 10)	0.01 ± 0.01 (*n* = 4)	1.01 ± 0.05 (*n* = 7)
	Kyn	0.18 ± 0.09 (*n* = 12)	0.93 ± 0.23 (*n* = 10)	-	
7.4	Trp	0.24 ± 0.07 (*n* = 6)	2.02 ± 0.19 (*n* = 6)	0.07 ± 0.03 (*n* = 4)	1.07 ± 0.02 (*n* = 6)
	Kyn	0.11 ± 0.08 (*n* = 6)	1.88 ± 0.18 (*n* = 6)	-	
9.0	Trp	0.69 ± 0.23 (*n* = 5)	2.32 ± 0.15 (*n* = 6)	0.10 ± 0.03 (*n* = 4)	1.17 ± 0.04 (*n* = 6)
	Kyn	0.52 ± 0.23 (*n* = 5)	1.99 ± 0.16 (*n* = 6)	-	

[1]pH of the receptor solution; the subdermal solution pH was always 7.4.
[2]Average flux between 5 and 6 hours.
[3]Trp/Kyn ratio of the average flux to the cathode between 5 and 6 hours of current application.

There were no significant differences in the anodal extraction fluxes of either compound as a function of receptor solution pH. However, there were significant differences between the cathodal fluxes of Kyn at pH 7.4 and pH 4 and between pH 9 and pH 4. Similar results were observed for Trp. The data are summarized in Table S3.

Table S3. Comparison between anode and cathode flux after 6 h of reverse iontophoresis obtained at different receptor solution pH (ANOVA + Tukey's multiple comparison test). The significance levels: * $p < 0.05$, ** $p < 0.01$, *** $p < 0.001$.

pH	Kynurenine at the anode			Kynurenine at the cathode		
	t value	Pr(>|t|)	Significance	t value	Pr(>|t|)	Significance
7.4 − 4.0 == 0	−0.37	0.93	n.s.	2.99	< 0.05	*
9.0 − 4.0 == 0	1.91	0.16	n.s.	3.32	< 0.01	**
9.0 − 7.4 == 0	1.99	0.14	n.s.	0.30	0.95	n.s.
**pH**	**Tryptophan at the anode**			**Tryptophan at the cathode**		
	t value	Pr(>|t|)	Significance	t value	Pr(>|t|)	Significance
7.4 − 4.0 == 0	−0.54	0.85	n.s.	3.49	< 0.01	**
9.0 − 4.0 == 0	1.62	0.26	n.s.	4.52	< 0.001	***
9.0 − 7.4 == 0	1.87	0.17	n.s.	0.93	0.63	n.s.

5. Endogenous Trp and Kyn

The cumulative extraction of endogenous Trp by reverse iontophoresis at pH 7.4 is shown in Figure S9.

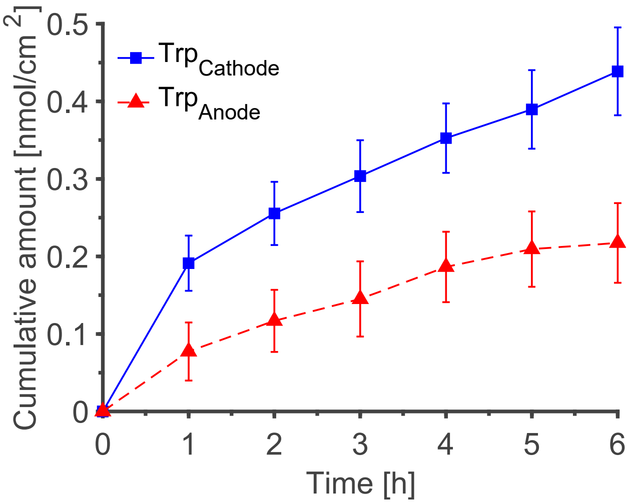

Figure S9. The cumulative amount of endogenous Trp extracted by reverse iontophoresis over 6 hours. The error bars represent mean ± SEM for n = 6

6. Post-iontophoretic Passive Extraction of Trp and Kyn

The cumulative amounts of Trp and Kyn extracted post-iontophoretically passive and compared with the corresponding passive controls are shown in Figure S10 and Table S4.

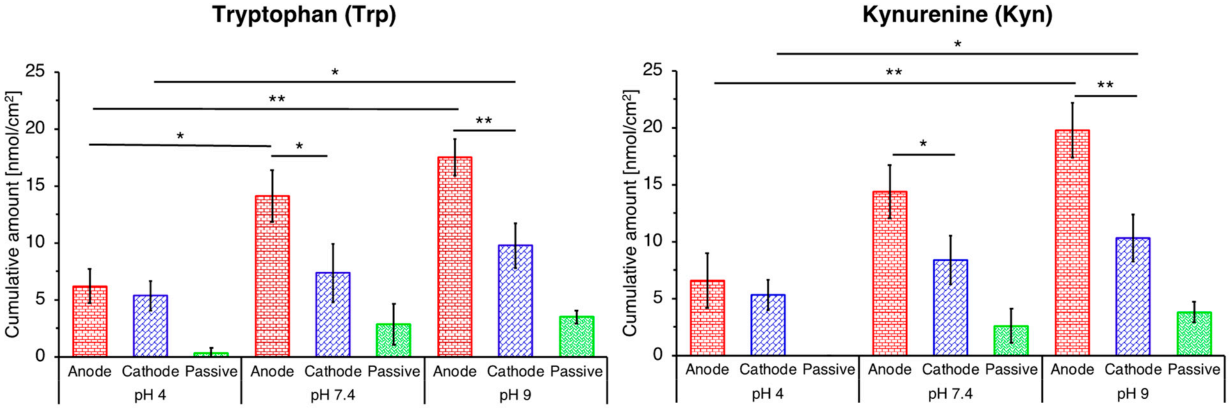

Figure S10. The cumulative amounts of Trp and Kyn extracted during post-iontophoresis at the anode (red), at the cathode (blue) and passively (green). The data presented are mean ± SEM. At pH 4, n_{anode} = 11, $n_{cathode}$ = 10, $n_{passive}$ = 4; at pH 7.4, n_{anode} = 6, $n_{cathode}$ = 6, $n_{passive}$ = 4; and at pH 9, n_{anode} = 5, $n_{cathode}$ = 6, $n_{passive}$ = 4. The significance levels: * $p < 0.05$, ** $p < 0.01$, *** $p < 0.001$.

Table S4. Amounts of Trp and Kyn 'released' from the skin in the 18-h period following 6 h of reverse iontophoretic or passive extraction. Data presented are mean ± SEM.

pH	Analyte	Cumulative Amount [nmol/cm²]			Trp/Kyn ratio		
		at anode	at cathode	Passive	at anode	at cathode	Passive
4.0	Trp	6.2 ± 1.5 (n = 11)	5.4 ± 1.3 (n = 10)	0.5 ± 0.5 (n = 4)	1.2 ± 0.2 (n = 10)	1.1 ± 0.03 (n = 9)	1.8 (n = 1)
	Kyn	6.6 ± 2.4 (n = 11)	5.3 ± 1.4 (n = 10)	0.3 ± 0.3 (n = 4)			
7.4	Trp	14.1 ± 2.3 (n = 6)	7.4 ± 2.6 (n = 6)	2.9 ± 1.7 (n = 4)	1.0 ± 0.02 (n = 6)	0.7 ± 0.1 (n = 6)	1.3 (n = 2)
	Kyn	14.4 ± 2.3 (n = 6)	8.4 ± 2.1 (n = 6)	2.6 ± 1.5 (n = 4)			
9.0	Trp	17.5 ± 1.6	9.8 ± 2.0	3.5 ± 0.5	0.9 ± 0.02	1.0 ± 0.02	1.2 ± 0.2

	(n = 5)	(n = 6)	(n =4)	(n = 5)	(n = 6)	(n = 4)
Kyn	19.9 ± 2.5<br>(n = 5)	10.3 ± 2.1<br>(n = 6)	3.8 ± 0.9<br>(n = 4)			

Statistical comparisons are collected in Table S5 and Table S6.

Table S5. Comparison between the post-iontophoresis amounts of Trp and Kyn 'released' into the anode and cathode receptor solutions at different pHs (ANOVA + Tukey's multiple comparison test). The significance levels: . $p < 0.1$, * $p < 0.05$, ** $p < 0.01$, *** $p < 0.001$.

**pH**	**Kynurenine at the Anode**			**Kynurenine at the Cathode**		
	**t value**	**Pr(>|t|)**	**Significance**	**t value**	**Pr(>|t|)**	**Significance**
7.4 − 4.0 == 0	2.25	0.09	.	1.73	0.22	n.s.
9.0 − 4.0 == 0	3.60	< 0.01	**	2.82	< 0.05	*
9.0 − 7.4 == 0	1.32	0.40	n.s.	0.98	0.60	n.s.
pH	Tryptophan at the anode			Tryptophan at the cathode		
	t value	Pr(>|t|)	Significance	t value	Pr(>|t|)	Significance
7.4 − 4.0 == 0	3.22	0.01	*	1.18	0.48	n.s.
9.0 − 4.0 == 0	4.33	< 0.01	**	2.57	< 0.05	*
9.0 − 7.4 == 0	1.16	0.49	n.s.	1.25	0.44	n.s.

Table S6. Post-iontophoresis anode-to-cathode (A/C) comparisons in the amounts of Trp and Kyn 'released' and their corresponding fluxes at different pHs (Two-tailed Student's t-test). The significance levels: * $p < 0.05$, ** $p < 0.01$, *** $p < 0.001$.

**pH**	**Post-iontophoretic cumulative amount**				**Post-iontophoretic flux**			
	Site	Analyte	*p*-value	Significance	Site	Analyte	*p*-value	Significance
4	A/C	Kyn	0.67	n.s.	A/C	Kyn	0.67	n.s.
	A/C	Trp	0.67	n.s.	A/C	Trp	0.67	n.s.
7.4	A/C	Kyn	< 0.05	*	A/C	Kyn	< 0.05	*
	A/C	Trp	< 0.05	*	A/C	Trp	< 0.05	*
9	A/C	Kyn	< 0.01	**	A/C	Kyn	< 0.01	**
	A/C	Trp	< 0.01	**	A/C	Trp	< 0.01	**

7. Reverse Iontophoretic Extraction Into a Bicontinuous Cubic Matrix

The results are summarized in Table S7.

Table S7. Cumulative amounts of Trp and Kyn extracted by reverse iontophoresis into a cubic phase and the corresponding Trp/Kyn ratios.

**Analyte**	**Cumulative Amount Extracted [nmol/cm^2]**				**Trp/Kyn Ratio**	
	Cubic at anode	Cubic at cathode	Total at anode	Total at cathode	at cathode cubic	at cathode total
Trp	0.68 ± 0.05<br>(n = 3)	5.3 ± 0.48<br>(n = 5)	0.68 ± 0.05<br>(n = 3)	6.6 ± 1.0<br>(n = 5)	0.94 ± 0.04<br>(n = 5)	0.86 ± 0.05<br>(n = 5)
Kyn	0.28 ± 0.28<br>(n = 3)	5.6 ± 0.45<br>(n = 5)	0.28 ± 0.28<br>(n = 3)	7.5 ± 0.83<br>(n = 5)		

The stability of the cubic phase to the reverse iontophoresis procedure was assessed by SAXD. The resulting diffraction patterns are shown in Figure S11.

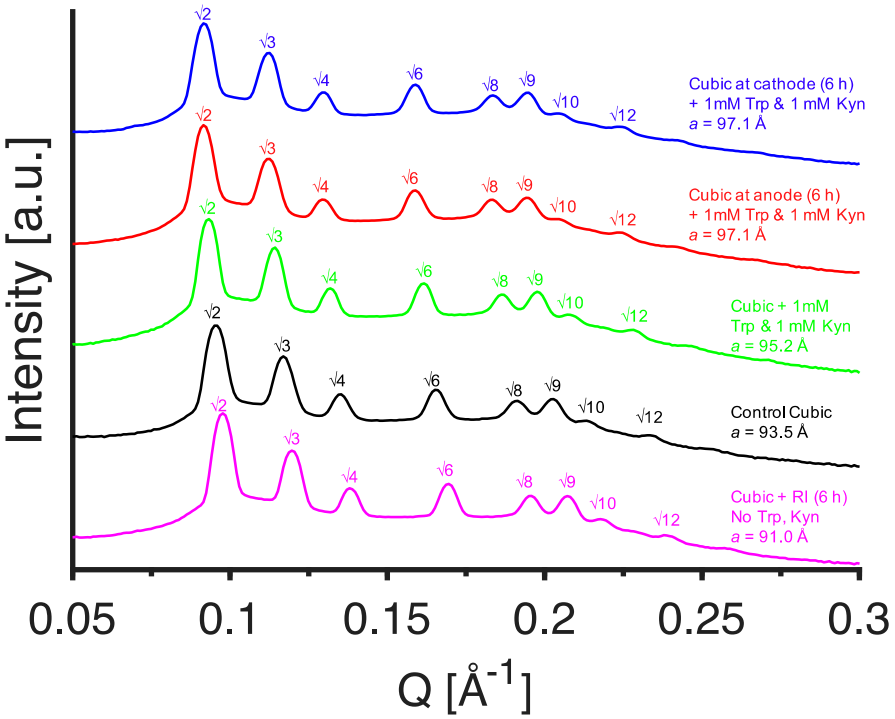

Figure S11. X-ray diffraction pattern of a fully swollen cubic phase (Pn3m) of GMO/H_2O without addition of Trp or Kyn (black line). Offset along the ordinate are additional X-Ray diffractograms of the cubic 'receiver' phase after 6 h of iontophoresis (0.5 mA/cm^2, magenta), equilibrated with 1 mM Trp and 1 mM Kyn (green) and after 6 h of reverse iontophoretic extraction of Trp and Kyn at the anode (red) and at the cathode (blue).

8. The Trp/Kyn Ratio

The ratios of the amounts and of the fluxes of Trp and Kyn extracted at the cathode by 6 h of reverse iontophoresis are shown in Figure S12.

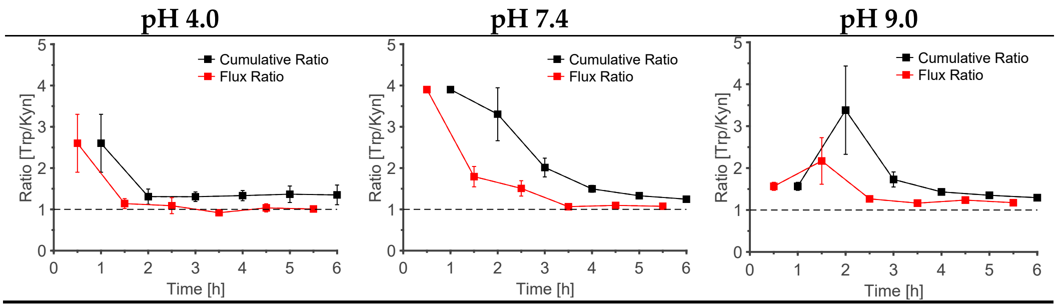

Figure S12. Trp/Kyn ratios at the cathode determined from the cumulative amounts (black) and corresponding flux (red) at each sampling point. Each data point presented is the mean ± SEM (pH 4.0 *n* = 3 for the first hour, *n* = 5 for second hour, *n* = 7 for third hour, *n* = 8 for fourth and fifth hours and *n* = 9 for sixth hour; pH 7.4: *n* = 1 for the first hour, *n* = 4 for the second hour and *n* = 6 for the rest; pH 9.0: *n* = 2 for the first hour and n = 6 for the rest). Dashed lines indicate the initial subdermal Trp/Kyn ratio.
